# Profiles of Work Engagement and Work-Related Effort and Reward Among Teachers: Associations to Occupational Well-Being and Leader–Follower Relationship During the COVID-19 Pandemic

**DOI:** 10.3389/fpsyg.2022.861300

**Published:** 2022-05-12

**Authors:** Sanni Pöysä, Eija Pakarinen, Marja-Kristiina Lerkkanen

**Affiliations:** ^1^Department of Teacher Education, University of Jyväskylä, Jyväskylä, Finland; ^2^Norwegian Centre for Learning Environment and Behavioural Research in Education, University of Stavanger, Stavanger, Norway

**Keywords:** work engagement, effort and reward, occupational well-being, work meaningfulness, leader–follower relationship

## Abstract

This study examined teachers’ occupational well-being by identifying profiles based on teachers’ self-ratings of work engagement as well as work-related effort and reward. It also did so by examining whether the identified subgroups differed with respect to teachers’ self-reported occupational stress and emotional exhaustion as well as with respect to work-related resources such as the individual resource of work meaningfulness and the leader-level resource of the leader–follower relationship. The participants in the study were 321 Finnish elementary school teachers. The data were collected in spring 2021, that is, at the time when the COVID-19 pandemic was present, yet there were no national school closures. Three groups of teachers were identified with latent profile analysis: (1) teachers recognized as being poorly engaged with the highest effort and lowest reward (4.7%); (2) teachers recognized as being averagely engaged with higher effort than reward (32.1%); and (3) teachers recognized as being highly engaged with higher reward than effort (63.2%). The subsequent analyses examining the differences among the profile groups revealed, for example, that each profile group differed with respect to the individual resource of work meaningfulness and profile groups 2 and 3 differed with respect to the leader-level resource of the leader–follower relationship. Thus, the findings indicate that there are differences in the ways in which teachers are able to benefit from the work-related resources and how they cope with job-related demands during the COVID-19 pandemic.

## Introduction

Teaching is known to be a highly demanding job (Kyriacou, 2001; Johnson et al., 2005), and there has been an increasing interest in teachers’ occupational well-being in educational research, especially during the COVID-19 pandemic. The existing literature has shown that occupational well-being has both positive and negative aspects (e.g., Bermejo-Toro et al., 2016; Cumming, 2017). Different negative aspects, such as experiences of work-related stress and emotional exhaustion, diminish teachers’ occupational well-being (e.g., Montgomery and Rupp, 2005; Foley and Murphy, 2015), while positive aspects, such as experiences of work engagement or work-related rewards, may strengthen their well-being (e.g., Van Vegchel et al., 2005; Bakker et al., 2007). However, as the existing studies have highlighted that teachers’ occupational well-being is individually constructed (e.g., Herman et al., 2018; Aulén et al., 2021), there are differences in the ways in which teachers’ well-being is built on the basis of different negative and positive aspects of occupational well-being.

Based on previous literature, a balance between work-related effort and reward, as well as between demands and resources, is particularly important for a sense of occupational well-being (e.g., Van Vegchel et al., 2005; Hakanen et al., 2006). This balance is perhaps not easily achieved or maintained as teachers’ work is determined by inherent changes that occur, for example, in myriad social interactions central to the occupation. Moreover, the last few years with the COVID-19 pandemic have shown that the educational field may also face such unexpected and relatively massive changes (UNESCO, UNICEF, and the World Bank, 2020), which may have an effect on teachers’ occupational well-being (e.g., Chan et al., 2021). In order to gain a deeper understanding on teachers’ occupational well-being, there is an evident need to examine the possible differences in their well-being during the COVID-19 pandemic. Such understanding would help teachers, researchers, and policy makers to better prepare for the future crises and other unexpected changes which can undermine teachers’ occupational well-being. For example, the knowledge gained with respect to different resources that could be endorsed as a source of focused support for teachers’ occupational well-being is important for both, practice and theory, during the pandemics and beyond.

To address the gaps in the previous literature, the present study was conducted by recognizing that teachers’ occupational well-being is individually constructed and by examining both positive and negative aspects related to occupational well-being simultaneously with the data collected during the COVID-19 pandemic. A person-oriented approach was utilized to identify subgroups of teachers based on their experiences of work engagement and work-related effort and reward. The subgroups were subsequently analyzed to examine whether they would differ, for example, with respect to teachers’ self-reported occupational stress and emotional exhaustion and with respect to teachers’ experience of their work meaningfulness or leader–follower relationships.

## Teachers’ Work Engagement and Occupational Well-Being

Across different occupations, the experience of being engaged with work is seen to be positively related to the experiences of occupational well-being (e.g., Schaufeli and Taris, 2014). This could be explained with the view of Schaufeli et al. (2006) that engaged employees have a sense of being effectively and energetically connected with their work-related activities, and they have a sense of ability to deal with their work-related demands. While the concept of work engagement can be approached *via* several different types of conceptualizations (see Christian et al., 2011; Perera et al., 2018), the definition that is perhaps the most acknowledged is drawn by Schaufeli et al. (2002). It defines work engagement as “a positive, fulfilling, work-related state of mind that is characterized by vigor, dedication, and absorption” (74). Based on previous literature (Schaufeli et al., 2002; Schaufeli and Salanova, 2013), teachers’ experience of vigor means that they have high levels of energy and mental resilience while working, are willing to invest effort in their work, and are persistent in the face of difficulties. Teachers’ experience of dedication, in turn, means that they are strongly involved with their work, and they have a sense of significance, enthusiasm, inspiration, pride, and challenge with respect to their work. Finally, teachers’ experience of absorption means that they are fully concentrated and happily engrossed in their work in such a way that time passes quickly while working.

According to the Job Demands-Resources (JD-R) Model (Demerouti et al., 2001), an experience of work engagement is built on the presence of job-related resources (see, e.g., Schaufeli and Taris, 2014; Lesener et al., 2020). The JD-R model does not determine what the resources specifically are, but previous literature has suggested that different resources can be identified within the individual or at multiple levels of the organization (i.e., group level, leader level, and organizational level; e.g., Nielsen et al., 2017). Individual resources are, for example, personal characteristics, such as self-efficacy and competence, or a sense of work’s meaningfulness, which helps a worker to cope with the demands of the job (Geldenhuys et al., 2014; Nielsen et al., 2017; Minkkinen et al., 2020). Group-level and leader-level resources, in turn, are different forms of social capital embedded in relationships among actors, as group-level resources are, for example, social support and good relationships between employees, while leader-level resources are, for example, leadership characteristics and the quality of leader–follower relationships (Christian et al., 2011; Nielsen et al., 2017; Lesener et al., 2020). The organizational-level resources are, for example, the way in which the work is organized, designed, and managed (Nielsen et al., 2017). Resources related to each of these three levels of organization are known to predict work engagement over time (Lesener et al., 2020).

Previous research has recognized different job resources, such as experiences of self-efficacy, supervisory support, and supportive colleagues, which are connected to teachers’ work engagement (e.g., Hakanen et al., 2006; Simbula et al., 2010). Such resources are important because they not only boost teachers’ work engagement but are also positively related to their occupational well-being in general (e.g., Collie and Martin, 2017). From the perspective of the JD-R model, occupational well-being is seen as drawing upon a balance between positive and negative job characteristics, where the positive job characteristics are different job resources, while the negative job characteristics are the demands that are typical for a specific occupation (Schaufeli and Taris, 2014). Based on previous research, the demands within the teaching profession include, for example, experiences of time pressure and excessive workloads (Hakanen et al., 2006; Skaalvik and Skaalvik, 2018).

Along with its role in the JD-R model, a balance between job-related demands and resources is acknowledged as crucial for occupational well-being in other theoretical models as well. The Effort–Reward Imbalance (ERI) model (Siegrist, 1996; Siegrist et al., 2004), for instance, posits that an imbalance between high effort and low reward may lead to increased work stress and decreased occupational well-being. In the ERI model, effort is seen to be constituted of extrinsic demands of the job and an individual’s intrinsic motivations in demanding situations (Siegrist, 1996), that is, different job demands and obligations that are imposed upon an individual (Van Vegchel et al., 2005). Reward, in turn, captures occupational gratifications in terms of salary, esteem, and career opportunities (Siegrist, 1996). Previous research examining teachers’ occupational well-being by using measures operationalized on the basis of the ERI model determined that teachers’ experiences of high levels of effort and low levels of reward as well as an effort–reward imbalance are related to their experiences of burnout (Unterbrink et al., 2007; Wang et al., 2015) as well as with their decreased physical (Bellingrath et al., 2010) and mental health (Hinz et al., 2014).

The previous literature shows that teachers’ occupational well-being is an indisputably complex phenomenon. Both theoretical models and empirical evidence indicate that occupational well-being is drawn from different positive and negative aspects (e.g., Van Vegchel et al., 2005; Schaufeli and Taris, 2014; Bermejo-Toro et al., 2016). For example, teaching is recognized as a highly stressful occupation (Kyriacou, 2001; Johnson et al., 2005), and when compared with other occupations, teachers’ stress seems to be higher than average (Johnson et al., 2005; Travers, 2017). However, despite being a highly stressful occupation, teachers’ work engagement was found to be relatively high even when measured during the first few months of the COVID-19 pandemic (e.g., Salmela-Aro et al., 2019; Pöysä et al., 2021). Thus, it is reasonable to approach teachers’ occupational well-being by recognizing the simultaneous presence of different negative and positive aspects related to well-being during the ongoing pandemic. Moreover, as increasing evidence has indicated that teachers’ occupational well-being is individually constructed, it might be that the complexity of teachers’ occupational well-being leads to an evident need to adapt statistical methods that take such complexity into consideration and examine teachers without seeing them as a homogeneous group.

## Teachers’ Occupational Well-Being During the COVID-19 Pandemic

In spring 2020, the COVID-19 pandemic resulted in unexpected and severe disruptions in the field of education. Since the beginning of the pandemic, there have been school closures around the globe, and by the end of September 2021, there were up to 27% of countries where the schools were still fully or partially closed (UNICEF, 2021). While Finland and many other Western countries were able to keep the schools mostly open or at least partially open in the academic year of 2020–2021 (UNESCO, 2021), the pandemic resulted in certain restrictions that affected daily schooling (Ministry of Education and Finnish Institute for Health and Welfare, 2020; see also UNESCO, UNICEF, the World Bank, the World Food Programme, and UNHCR, 2021). Schools have been guided, for example, to ensure that there are no unnecessary physical contacts between teachers and between students (Ministry of Education and Finnish Institute for Health and Welfare, 2020). In addition, teachers and students have not been allowed to go to school if they have even the slightest symptoms of flu (Ministry of Education and Finnish Institute for Health and Welfare, 2020); thus, teachers have needed to support the learning of both students at school and those at home.

The field of education has shared a reasonable concern about teachers’ occupational well-being during the COVID-19 pandemic. The number of studies examining teachers’ well-being at the time of school closures has been increasing. Most of the existing findings thus far have suggested that teachers experienced substantial levels of occupational stress during the spring of 2020 (e.g., MacIntyre et al., 2020), yet there are also some contradictory findings (Herman et al., 2021). Nevertheless, based on the prevalence of acute stress symptoms, Zhou and Yao (2020) suggested that teachers had relatively serious acute stress symptoms at the beginning of the pandemic, and, for example, Chan et al. (2021) and Pöysä et al. (2021) have shown that teachers’ experiences of occupational stress were positively related to their emotional exhaustion during that time. Moreover, previous studies have recognized that the beginning of the pandemic resulted in stressors specifically related to the situation (e.g., Kim and Asbury, 2020; MacIntyre et al., 2020); thus, it is possible that teachers’ experiences of job-related demands increased due to the COVID-19 pandemic.

Along with focusing on the negative aspects related to occupational well-being, the existing literature provides some suggestions as to which factors may have supported teachers’ well-being at the time of school closures. Such factors can be recognized with respect to individuals as well as to different levels of organization. For example, Herman et al. (2021) found that teachers’ sense of confidence in managing student behavior in online settings predicted teachers’ stress negatively. MacIntyre et al. (2020), in turn, have found that teachers’ usage of approach coping strategies (i.e., strategies that aim to change or actively accept the stressor) was related to positive well-being experiences, while usage of avoidant coping strategies was related to stress and other negative emotions. The existing literature does not provide a view on how teachers’ individual resources of work meaningfulness could have been related to teachers’ occupational well-being during the COVID-19 pandemic. However, based on a study by Minkkinen et al. (2020) that suggested that a sense of meaningful work protects teachers’ well-being under stressors, it could be expected that a sense of work’s meaningfulness might have been crucial, especially during the pandemic.

With respect to different levels of organization, social support of colleagues (i.e., group-level resource) and support gained from a leader (i.e., leader-level resource) are recognized as important predictors of teachers’ occupational well-being in the time of school closures. For example, Spicksley et al. (2021) found that teachers who experienced being supported by other teachers in their school felt more able to cope with the challenges resulted from school closures due to the COVID-19 pandemic. Collie (2021), in turn, examined the role of principal leadership and workplace buoyancy in teachers’ occupational well-being during the spring of 2020. She found that autonomy-thwarting leadership was related to teachers’ increased experiences of emotional exhaustion, while autonomy-supportive leadership increased workplace buoyancy, which in turn decreased teachers’ somatic burden, stress, and emotional exhaustion.

While most of the prior findings have been reached by utilizing a traditional variable-oriented approach, the existing literature also consists of some rare studies in which teachers’ occupational well-being during the time of school closures has been examined using a person-oriented approach. For example, Pöysä et al. (2021) have identified profiles of teachers based on their occupational stress and work engagement, and Salmela-Aro et al. (2020) have identified profiles based on teachers’ work-related burnout and work engagement, both studies based on the data that had been collected in spring 2020. The findings suggested that there were differences in levels of stress (Pöysä et al., 2021) and levels of burnout (Salmela-Aro et al., 2020) along with differences in levels of work engagement between the profile groups. Thus, even at the time of school closures and at the beginning of the COVID-19 pandemic, teachers were not unanimous with respect to their occupational well-being.

Unfortunately, strains that the COVID-19 pandemic caused to teachers’ occupational well-being did not end at the time when full closures of schools ended. As long as the pandemic is present, it continues to affect daily schooling as well. For example, in Finland, teachers have reported that hybrid teaching, that is, a situation where a number of students are participating remotely while others are at school, is not ideal for the sake of teachers or their students (Vuorio et al., 2021). More research is needed to examine teachers’ occupational well-being at the time when the pandemic is present, yet schools are open. That kind of knowledge would be particularly helpful when considering the possibility of future pandemics and the well-being of teachers during the pandemics and beyond.

## The Present Study

The aim of the present study was to examine teachers’ occupational well-being at the time when the COVID-19 pandemic was present, yet teachers and students were mostly at school, but there were some national or local restrictions that teachers had to follow. Based on the previous findings (e.g., Salmela-Aro et al., 2020; Aulén et al., 2021; Pöysä et al., 2021), the person-oriented approach was chosen to acknowledge that teachers’ occupational well-being has been individually constructed before and during pandemic. Moreover, to appreciate the view that occupational well-being is drawn from both negative and positive aspects (Van Vegchel et al., 2005; Schaufeli and Taris, 2014), the presence of subgroups was examined based on teachers’ experiences of work engagement as well as work-related effort and reward. In addition, to obtain more detailed knowledge, subsequent analyses were conducted. Those examined whether the subgroups would differ with respect to teachers’ self-reported occupational well-being (i.e., occupational stress and emotional exhaustion) and self-reported changes in occupational well-being (i.e., occupational stress and emotional exhaustion) as well as change in work-related effort and reward due to the COVID-19 pandemic. Moreover, our subsequent analyses examined possible differences among the subgroups with respect to the teachers’ individual resource of work meaningfulness and the leader-level resource of leader–follower relationship. The following research questions and hypotheses were formulated:

RQ1. What kind of profile groups can be identified based on teachers’ experiences of work engagement (i.e., vigor, dedication, and absorption) and work-related effort and reward? Based on previous findings suggesting different profiles among teachers before the COVID-19 pandemic (Herman et al., 2018; Aulén et al., 2021) as well as at the beginning of the pandemic (Salmela-Aro et al., 2020; Pöysä et al., 2021), it was expected that several distinct subgroups would be identified in the present sample as well (Hypothesis 1).

RQ2. To what extent do the identified profile groups differ in teachers’ self-reported occupational well-being (i.e., occupational stress and emotional exhaustion) and in teachers’ self-reported change of occupational well-being (i.e., occupational stress and emotional exhaustion) as well as change in work-related effort and reward due to the COVID-19 pandemic? Based on previous literature indicating that teachers’ work engagement and work-related effort and reward are related to teachers’ occupational stress and experiences of emotional exhaustion (e.g., Unterbrink et al., 2007; Wang et al., 2015; Skaalvik and Skaalvik, 2016; Pöysä et al., 2021), it was expected that subgroups would differ with respect to teachers’ occupational well-being (Hypothesis 2a) as well as with respect to change of occupational well-being due to the COVID-19 pandemic (Hypothesis 2b). In addition, based on previous findings showing that there were certain job-related demands related to the time when the COVID-19 pandemic began (e.g., Kim and Asbury, 2020; MacIntyre et al., 2020), it was expected that subgroups would differ with respect to change in work-related effort and reward due to the COVID-19 pandemic (Hypothesis 2c).

RQ3. To what extent do the identified profile groups differ with respect to the individual resource of work meaningfulness and the leader-level resource of leader–follower relationship? Based on a previous study showing that a sense of meaningful work protects teachers’ well-being under stressors (Minkkinen et al., 2020), it was expected that the subgroups would differ based on the self-reported individual resource of work meaningfulness (Hypothesis 3a). In addition, based on previous findings showing positive relations between work engagement and the leader–follower relationship (Christian et al., 2011) as well as findings indicating the importance of supportive leadership on teachers’ occupational well-being (Collie, 2021), it was expected that the profile groups would differ based on the leader-level resource of the leader–follower relationship (Hypothesis 3b).

## Materials and Methods

### Participants and Procedure

The present study was conducted under a larger Teacher and Student Stress and Interaction in Classroom study (TESSI; Lerkkanen and Pakarinen, 2021). The ethical committee of the University of Jyväskylä provided ethical approval for the study, and the research was conducted following the national guidelines for the ethical principles of research with human participants (Finnish National Board on Research Integrity TENK, 2019). The permits to conduct the data collection were also requested and granted from local education authorities before contacting the teachers. The data for the present study were collected from several municipalities located in different areas of Finland in spring 2021. Teachers were approached *via* e-mail by asking whether they would agree to answer a questionnaire concerning their occupational well-being and teaching practices during the COVID-19 pandemic. Before answering the questionnaires, teachers were also asked to read the privacy notices of the study and to mark whether they were participating in the study freely and willingly.

The participants in the present sample were 321 teachers in Grades 1–6. A total of 77.6% of the participants were female and 21.5% male (0.9% chose the option “prefer not to answer”). The vast majority of the participants (98.8%) had a master’s degree in education and was qualified to work as teachers in elementary school. Participants’ ages ranged from 24 to 68 years (*M* = 46.3 years; *SD* = 9.8 years; *Mdn* = 46.5 years), and work experience ranged from 1 to 41 years (*M* = 17.4 years; *SD* = 10.3 years; *Mdn* = 17.0 years).

### Measures

#### Work Engagement and Work-Related Effort and Reward

The two main measures of the present study were the Utrecht Work Engagement Scale (UWES; Schaufeli et al., 2002; see also Seppälä et al., 2009) and the ERI scale (Siegrist et al., 2004; see also Rantanen et al., 2012). Teachers’ work engagement was measured with a nine-item version of the UWES measure. The three measured subscales were as: (1) vigor (3 items; *α* = 0.88; e.g., “At my work, I feel bursting with energy.”), (2) dedication (3 items, *α* = 0.90; e.g., “I am enthusiastic about my job.”), and (3) absorption (3 items, *α* = 0.88; e.g., “I feel happy when I am working intensely.”). The teachers were asked to answer the items on a 7-point Likert scale (1 = *never*; 7 = *daily*). The mean values for each subscale were calculated and used in further analyses. Based on the norm scores drawn across occupations (Schaufeli and Bakker, 2004), vigor is considered high when the average value for the subscale is 5.81–6.65, dedication is considered high when the average value is 5.71–6.69, and absorption is considered high when the average value is 5.21–6.33.

The ERI scale was used to measure teachers’ work-related effort and reward. The subscale of effort was measured with six items (*α* = 0.78; e.g., “I have constant time pressure due to a heavy work load.”) and the subscale of reward with five items (*α* = 0.75; e.g., “I experience adequate support in difficult situations.”). In the subscale of reward, the question concerning salary was excluded from the analyses based on Levene’s test. Teachers were asked to answer the items on a 4-point Likert scale (1 = *strongly disagree*; 4 = *strongly agree*), and a higher score represented higher effort and reward. The mean values for both subscales were calculated and used in analyses. The ERI scale has been widely used, and it has been found to be valid in different countries (Van Vegchel et al., 2005), including Finland (e.g., Kinnunen et al., 2008; Feldt et al., 2016).

#### Other Measures

##### Teachers’ Occupational Stress

The extent of teachers’ occupational stress was measured with the following question: “Stress means a situation in which a person feels tense, restless, nervous, or anxious, or is unable to sleep at night because his/her mind is troubled all the time. Do you feel this kind of stress these days?” (Elo et al., 2003). Teachers were asked to answer the item on a 6-point Likert scale (1 = *not at all*; 6 = *very much*). The previous literature has verified that this single-item measure is valid for identifying occupational stress (Elo et al., 2003; see also Eddy et al., 2019).

##### Emotional Exhaustion

The extent of teachers’ emotional exhaustion was measured using a shortened Finnish version of the Bergen Burnout Inventory (BBI; Salmela-Aro et al., 2011). The three items constituting the subscale of emotional exhaustion were used (*α* = 0.77; e.g., “I am snowed under with work”). Teachers were asked to answer on a 6-point Likert scale (1 = *completely disagree*; 6 = *completely agree*). The mean value for the subscale was calculated and used in analyses.

##### Changes in Occupational Well-Being Due to the COVID-19 Pandemic

To examine possible changes in teachers’ occupational well-being due to the COVID-19 pandemic, the present study used two measures created for this purpose. One of those measures, a single-item question measuring teachers’ occupational stress due to the COVID-19 pandemic, has been used previously (Pöysä et al., 2021). The single-item question rated on a 4-point Likert scale (1 = *not at all*; 4 = *entirely*) was “To what extent has the increase in your occupational stress been due to the COVID-19 situation?” The other measure including three items focused on change in emotional exhaustion. The measure was adapted by asking teachers to consider along of original measure for emotional exhaustion (i.e., the original subscale of emotional exhaustion of the Finnish version of the BBI; Salmela-Aro et al., 2011) whether their experiences had remained relatively the same or whether there was an increase or decrease with the experience that specific item focused on. The internal consistency for the adapted subscale was acceptable (*α* = 0.74), and the mean value for the subscale was calculated and used in analyses.

##### Changes in Work-Related Effort and Reward Due to the COVID-19 Pandemic

Teachers’ experiences of change with respect to work-related effort and reward due to the COVID-19 pandemic were measured with an adapted measure of the work-related effort and reward ERI scale (i.e., Siegrist et al., 2004). Teachers were asked to consider whether their experiences had remained relatively the same or whether there was an increase or decrease with the experience that specific item focused on. The internal consistencies for the adapted subscales were acceptable (six items for change in effort, *α* = 0.81; five items for change in reward, *α* = 0.69), and the mean values for both subscales were calculated and used in analyses.

##### Work Meaningfulness

The individual resource of the work’s meaningfulness was measured with the Work as Meaning Inventory (WAMI; Steger et al., 2012). The four items that constitute the subscale of positive meaning were used (*α* = 0.93; e.g., “I have a good sense of what makes my job meaningful.”). Teachers were asked to answer on a 5-point Likert scale (1 = *completely disagree*; 5 = *completely agree*). The mean value for the subscale was calculated and used in analyses.

##### Leader–Follower Relationship

The leader-level resource of the leader–follower relationship was measured with the Finnish version of the Leader–Member Exchange measure (LMX; Graen and Uhl-Bien, 1995; see also Norvapalo, 2014). Teachers were asked to answer seven items (*α* = 0.93; e.g., “My leader understands my work problems and needs.”) on a 5-point Likert scale (1 = *disagree*; 5 = *agree*). The mean value for the items was calculated and used in analyses.

### Statistical Analyses

A person-oriented approach with a latent profile analysis (LPA; Vermunt and Magidson, 2002; Lubke and Muthén, 2005) was utilized in the present study. LPA is a model-based variant of traditional cluster analysis that aims to identify the smallest number of latent classes that adequately describe the associations between observed continuous variables (Vermunt and Magidson, 2002; Nylund-Gibson and Masyn, 2016). The advantage of LPA is that it recognizes that populations are not necessarily heterogeneous in how the measured variables are related to possible outcomes (Bergman and Trost, 2006; Laursen and Hoff, 2006).

When conducting the enumeration process, a series of LPAs are performed to examine profile solutions that differ with respect to the number of profiles. The best-fitting solution is recognized based on the fit indices as well as theoretical and practical considerations. The fit indices used in the present study were log-likelihood (log L), Akaike information criterion (AIC), Bayesian information criterion (BIC), and adjusted Bayesian information criterion (ABIC) as well as the Vuong–Lo–Mendell–Rubin (VLMR) likelihood ratio test and the adjusted Lo–Mendell–Rubin (LMR) test. The LPA with the lowest log L, AIC, BIC, and ABIC values is considered to provide a good fit to the data, and *p* > 0.05 with VLMR and LMR indicates that the model with one less class should be rejected in favor of the estimated model (e.g., Lo et al., 2001; Nylund et al., 2007).

In the present study, LPAs were conducted using teachers’ self-ratings on their vigor, dedication, and absorption (i.e., three subscales of work engagement) as well as self-ratings of their work-related effort and reward. The LPAs were conducted using the Mplus statistical package (version 7.4; Muthén and Muthén, 1998). The subsequent analyses comparing the profile groups with the multinomial regression analyses and pairwise comparisons with respect to teachers’ occupational stress, emotional exhaustion, work meaningfulness, leader–follower relationship, and changes in occupational well-being during the COVID-19 pandemic were conducted for a best-fitting profile solution by utilizing the auxiliary function and the three-step procedure along with the LPA. To validate the chosen profile solution, one-way ANOVA and pairwise comparison were conducted using the IBM SPSS Statistics Version 26 in terms of the criterion variables.

## Results

### Descriptive Statistics

Descriptive statistics of the criterion variables on which the latent profile analysis was based (i.e., vigor, dedication, and absorption as well as the work-related effort and reward) are presented in Table 1. Based on descriptive statistics concerning teachers’ work engagement, the participating teachers experienced, on average, high levels of dedication and absorption, and average levels of vigor when compared with the norm scores across occupations (Schaufeli and Bakker, 2004). Descriptive statistics concerning teachers’ work-related effort and reward suggested that teachers experienced, on average, their work-related demands and rewards at somewhat similar levels. Furthermore, correlations calculated for the criterion variables (Table 1) suggested strong and somewhat strong positive correlations among the three subscales of work engagement (i.e., vigor, dedication, and absorption). In addition, a somewhat moderate negative correlation was found between work-related effort and reward. Correlations among the three subscales of work engagement and effort were negative, yet only weak at most. Instead, correlations among the three subscales of work engagement and reward were positive, but only weak or somewhat moderate.

**Table 1:** Descriptive statistics and correlation matrix for the criterion variables upon which the latent profile analysis was based.

	*M* (*SD*)	min	max	1	2	3	4	5
1. Vigor	5.68 (1.16)	1	7		0.89^**^	0.70^**^	−0.37^**^	0.41^**^
2. Dedication	5.87 (1.17)	1	7			0.73^**^	−0.30^**^	0.39^**^
3. Absorption	5.62 (1.27)	1	7				−0.13^*^	0.29^**^
4. Effort	2.92 (0.55)	1	4					−0.42^**^
5. Reward	2.96 (0.58)	1	4					

Work engagement constituting vigor, dedication, and absorption: 1 (never) to 7 (daily); Work-related effort and reward: 1 (strongly disagree) to 4 (strongly agree).

* *p* > 0.05;

** *p* > 0.01.

### The Identified Profile Groups

LPAs were conducted to examine what kind of profile groups, based on teachers’ experiences of work engagement (i.e., vigor, dedication, and absorption) and work-related effort and reward, could be identified. During the enumeration process, the results of LPAs demonstrated that the fit indices of log L, BIC, ABIC, and AIC decreased when the number of profiles increased without providing a point of elbowing (Table 2). The *p*-values (>0.05) for both the VLMR and LMR tests suggested that up to a three-profile solution, the model with one less profile could be rejected in favor of the estimated model. The three-profile solution, which provided the lowest *p*-values in the VLMR and LMR tests, was determined to provide the most optimal fit with the data, as it was also theoretically and practically reasonable.

**Table 2:** Fit indices for the series of latent profile analyses (LPAs).

Number of classes	Log L	AIC	BIC	ABIC	*p*VLMR	*p*LMR	*n*	Entropy
1	−2083.89	4187.78	4225.50	4193.78			321	
2	−1831.79	3695.57	3755.91	3705.16	0.016	0.017	86/235	0.895
**3**	**−1679.94**	**3403.88**	**3486.85**	**3417.07**	**0.002**	**0.002**	**15/103/203**	**0.931**
4	−1623.66	3303.32	3408.92	3320.11	0.629	0.634	15/55/93/158	0.864
5	−1576.23	3220.45	3348.68	3240.84	0.050	0.052	54/10/107/26/124	0.890
6	−1554.94	3189.89	3340.74	3213.87	0.350	0.357	10/52/23/8/126/102	0.888

Log L, Log-likelihood; AIC, Akaike information criterion; BIC, Bayesian information criterion; ABIC, adjusted Bayesian information criterion; VLMR, Vuong–Lo–Mendell–Rubin likelihood ratio test; and LMR, adjusted Lo–Mendell–Rubin test.

**Figure 1:**
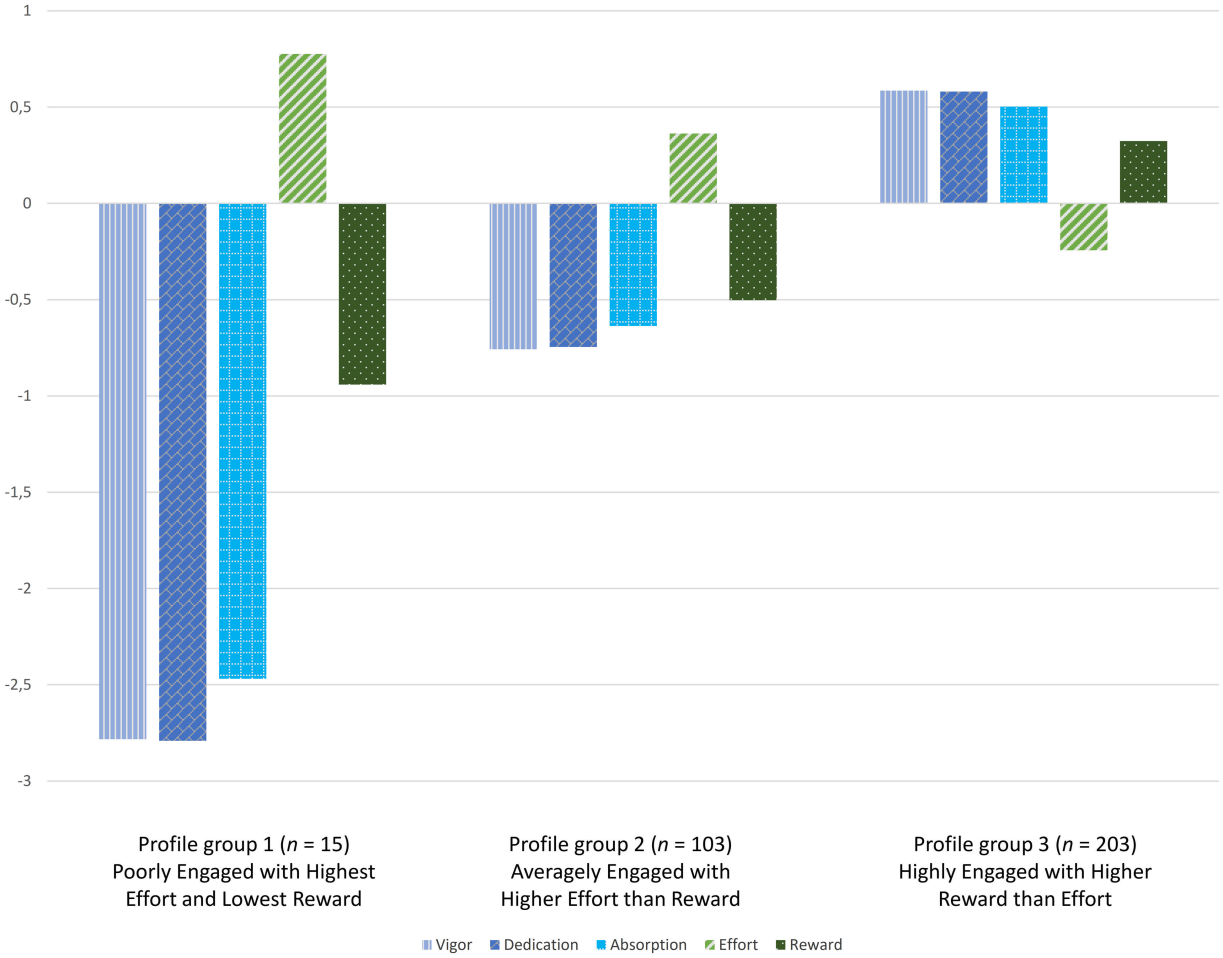
The three-profile groups based on work engagement and work-related effort and reward.

In the three-profile solution (Figure 1; Table 3), the first profile group comprised 4.7% (*n* = 15) of the participating teachers. Teachers in this profile group were found to have the lowest levels of vigor, dedication, and absorption, while they also had the highest level of effort and lowest level of reward. Thus, profile group 1 was named *Poorly Engaged with Highest Effort and Lowest Reward*. The second profile group comprised 32.1% of participating teachers (*n* = 103). Profile group 2 was composed of teachers whose vigor, dedication, and absorption were at average levels, and their experience of effort was higher than their experience of reward. Thus, profile group 2 was named *Averagely Engaged with Higher Effort than Reward*. The third profile group applied to 63.2% (*n* = 203) of the participating teachers. Teachers in this profile group were found to have high levels of vigor, dedication, and absorption, and their experience of reward was higher than their experience of effort. Thus, profile group 3 was named *Highly Engaged with Higher Reward than Effort*.

**Table 3:** Differences in work engagement and work-related effort and reward among profile groups.

	Profile group 1 Poorly Engaged with Highest Effort and Lowest Reward (*n* = 15)	Profile group 2 Averagely Engaged with Higher Effort than Reward (*n* = 103)	Profile group 3 Highly Engaged with Higher Reward than Effort (*n* = 203)	ANOVA	Pairwise comparison
	*M* (*SD*)	*M* (*SD*)	*M* (*SD*)	*F*(2, 317)	
Vigor	2.44 (0.73)	4.80 (0.68)	6.36 (0.48)	517.84^***^	^2^1 < 2, 3; 2 < 3
Dedication	2.60 (0.77)	5.00 (0.74)	6.55 (0.46)	496.77^***^	^2^1 < 2, 3; 2 < 3
Absorption	2.49 (1.27)	4.81 (1.07)	6.26 (0.63)	215.88^***^	^2^1 < 2, 3; 2 < 3
Effort	3.34 (0.36)	3.11 (0.49)	2.78 (0.56)	19.26^***^	^1^1, 2 > 3
Reward	2.41 (0.54)	2.67 (0.47)	3.15 (0.55)	37.15^***^	^1^1, 2 < 3

Work engagement constituting vigor, dedication, and absorption: 1 (never) to 7 (daily); Work-related effort and reward: 1 (strongly disagree) to 4 (strongly agree); Pairwise comparisons reported among groups in which differences were statistically significant at *p* < 0.001 with ANOVA post-hoc.

1 LSD.

2 Dunnett T3 correction.

*** *p* > 0.001.

The three-profile solution was validated with one-way ANOVAs and pairwise comparisons in terms of the criterion variables on which the LPA was based. The results of the ANOVAs suggested that all profiles differed from each other (Table 3). The results of pairwise comparisons complemented this by suggesting that each profile had its own unique features when compared with other profile groups.

### Differences in Teachers’ Well-Being Among the Profile Groups

Multinomial regression analyses and pairwise comparisons were conducted to examine the extent to which the identified profile groups differed in teachers’ self-reported occupational well-being (i.e., occupational stress and emotional exhaustion). The findings showed that the three identified profile groups differed to some extent with respect to occupational stress. Teachers in profile group 1 (i.e., *Poorly Engaged with Highest Effort and Lowest Reward*) reported significantly higher levels of occupational stress than teachers in profile group 2 (i.e., *Averagely Engaged with Higher Effort than Reward*; *β* = 0.68, *p* = 0.023) or in profile group 3 (i.e., *Highly Engaged with Higher Reward than Effort*; *β* = 1.02, *p* = 0.002). Profile groups 2 and 3 did not differ significantly from one another. In addition, with respect to teachers’ experiences of emotional exhaustion, the profile groups did not differ from one another.

To examine whether the profile groups would differ with respect to self-reported changes in teachers’ occupational well-being due to the COVID-19 pandemic, multinomial regression analyses and pairwise comparisons were also conducted for the measures that were adapted for this purpose. First, the results concerning the single-item question focusing on the extent to which the increase in their occupational stress was related to the COVID-19 situation suggested that teachers in profile group 3 (i.e., *Highly Engaged with Higher Reward than Effort*) experienced that the COVID-19 pandemic had less to do with their possible increase of occupational stress than teachers in profile group 1 (i.e., *Poorly Engaged with Highest Effort and Lowest Reward*; *β* = −1.36, *p* = 0.006) or in profile group 2 (i.e., *Averagely Engaged with Higher Effort than Reward*; *β* = −0.58, *p* = 0.026). Profile groups 1 and 2 did not differ significantly from one another.

Second, the results concerning the change in teachers’ emotional exhaustion suggested that teachers in profile group 3 (i.e., *Highly Engaged with Higher Reward than Effort*) reported that the COVID-19 pandemic resulted in less increase in their emotional exhaustion than teachers in profile group 1 (i.e., *Poorly Engaged with Highest Effort and Lowest Reward; β* = −2.05*, p* = *0.*042) or in profile group 2 (i.e., *Averagely Engaged with Higher Effort than Reward*; *β* = −1.48, *p* = 0.005). Profile groups 1 and 2 did not differ significantly from one another.

The results concerning work-related effort suggested that teachers in profile group 3 (i.e., *Highly Engaged with Higher Reward than Effort*) reported that the COVID-19 pandemic had resulted in a higher increase in their effort than teachers in profile group 2 (i.e., *Averagely Engaged with Higher Effort than Reward*; *β* = 1.42, *p* = 0.036). No other significant differences with respect to changes in effort were found among the profile groups. In addition, the results suggested that the profile groups did not differ with respect to changes in work-related rewards due to the COVID-19 pandemic.

### Differences in the Individual and Leader-Level Resources Among the Profile Groups

With respect to self-reported work meaningfulness, the results of multinomial regression analyses and pairwise comparisons showed that each profile group differed from the others. Teachers in profile group 1 (i.e., *Poorly Engaged with Highest Effort and Lowest Reward*) reported significantly lower levels in their work meaningfulness than teachers in profile group 2 (i.e., *Averagely Engaged with Higher Effort than Reward*; *β* = −1.32, *p* = 0.003) or in profile group 3 (i.e., *Highly Engaged with Higher Reward than Effort*; *β* = −4.16, *p* < 0.001). In addition, teachers in profile group 2 (i.e., *Averagely Engaged with Higher Effort than Reward*) reported a significantly lower level in their work meaningfulness than teachers in profile group 3 (i.e., *Highly Engaged with Higher Reward than Effort*; *β* = −2.84, *p* < 0.001).

Multinomial regression analyses and pairwise comparisons were also conducted to examine whether the profile groups would differ with respect to teachers’ self-reported experiences of their leader–follower relationship. The findings showed a significant difference between two profile groups. Teachers in profile group 3 (i.e., *Highly Engaged with Higher Reward than Effort*) reported a significantly higher quality of the leader–follower relationship than teachers in profile group 2 (i.e., *Averagely Engaged with Higher Effort than Reward*; *β* = 0.80, *p* = 0.001).

## Discussion

The present study contributes to the existing literature by supporting the view that teachers’ occupational well-being is individually constructed, and by identifying subgroups of teachers with different profiles related to occupational well-being during the time when the COVID-19 pandemic was present but there were no national school closures. The findings of this study contribute to the literature by suggesting that the identified profile groups differ to some extent, for example, with respect to teachers’ occupational stress as well as with respect to teachers’ individual resource of work meaningfulness and the leader-level resource of the leader–follower relationship. The knowledge gained in this study is valuable for teachers, researchers as well as policy makers for preparing the possible future crises. It is important to find ways to deal with the strains that possible pandemics and other unexpected changes could have on teachers’ occupational well-being.

First, as expected (Hypothesis 1), distinct subgroups were identified based on teachers’ experiences of work engagement (i.e., vigor, dedication, and absorption) and work-related effort and reward. Within the present three-profile solution, the smallest profile group was the one where teachers were recognized as being relatively *Poorly Engaged with Highest Effort and Lowest Reward* (Profile group 1; 4.7% of participants). Approximately one-third of the participating teachers were recognized as being *Averagely Engaged with Higher Effort than Reward* (Profile group 2; 32.1% of participants), and well above half of the participating teachers were recognized as being *Highly Engaged with Higher Reward than Effort* (Profile group 3; 63.2% of participants). The size of the smallest profile group (Profile group 1) was somewhat smaller than could have been expected based on previous studies. However, the existing studies from the beginning of the pandemic have identified profile groups, for example, based on work engagement and occupational stress (Pöysä et al., 2021) or based on work engagement and emotional exhaustion (Salmela-Aro et al., 2020), and there are no prior studies in which the profile solutions would have been examined with the same set of criterion variables that were used in the current study. In addition, it should be noted that profile group 1 (i.e., *Poorly Engaged with Highest Effort and Lowest Reward*) and profile group 2 (i.e., *Averagely Engaged with Higher Effort than Reward*) did not differ significantly with respect to work-related effort and reward. Thus, the smallest profile group consisted specifically of those teachers who were particularly poorly engaged.

According to previous literature, an experience of work engagement is built on the presence of job-related resources (e.g., Schaufeli and Taris, 2014; Lesener et al., 2020). In the present study, the criterion variables used for the latent profile analyses included teachers’ self-ratings of their work-related rewards. The items used tapped into teachers’ own experiences of receiving adequate support and appreciation along with having pleasant prospects for the future (Siegrist et al., 2004), and such items can also be seen as being related to job-related resources (see Nielsen et al., 2017; Lesener et al., 2020). The present findings concerning the largest profile group (i.e., profile group 3; *Highly Engaged with Higher Reward than Effort*) can be seen to be in congruence with the view that teachers’ work engagement and experience of job-related resources are related as: teachers who had the highest levels of work-related reward were also those with the highest levels of work engagement. However, as the other two profile groups differed significantly with respect to work engagement, yet not with respect to work-related reward, it can be assumed that, at least among some teachers, a level of work-related resources is not necessarily related to the level of work engagement. While the data used in the present study do not allow one to examine this further, it seems that, to some extent, an experience of work engagement can be built on the basis of job-related resources, but at the same time, it seems that at least lower levels of job-related resources do not determine the level of work engagement. Nevertheless, such findings can be seen as verifying previous findings (e.g., Salmela-Aro et al., 2020; Pöysä et al., 2021), indicating that there are differences in the ways in which teachers’ occupational well-being is construed. Therefore, the present findings further highlight that it is important to utilize analytical approaches that move beyond traditional variable-oriented approaches.

Second, Hypothesis 2a concerning the differences in teachers’ self-reported occupational well-being (i.e., occupational stress and emotional exhaustion) among the profile groups was only partially confirmed. As Hypothesis 2a predicted, the findings with respect to occupational stress suggest that teachers recognized as being *Poorly Engaged with Highest Effort and Lowest Reward* (i.e., Profile group 1) experienced occupational stress more than teachers in the other two profile groups did. However, teachers within the two other profile groups (i.e., profile groups 2 and 3) did not differ significantly. Thus, in the present sample, the prevalence of occupational stress seemed to be higher, especially among those teachers who were poorly engaged. While the lack of difference between profile groups 2 and 3 was somewhat surprising, the findings with respect to profile group 1 indicate that the experience of work engagement may provide protection against the elements, such as stress, that are damaging to occupational well-being. Such suggestions have been made in previous literature as well (e.g., Bermejo-Toro et al., 2016). Moreover, based on the present findings, it seems that in a sense of protecting oneself from stress, average level of work engagement is perhaps enough.

With respect to teachers’ experiences of emotional exhaustion, in contrast to what was expected (Hypothesis 2a), there were no statistically significant differences between the profile groups. This was astonishing, as a number of previous studies have found that teachers’ emotional exhaustion and burnout are negatively related to work engagement (e.g., Hakanen et al., 2006; Skaalvik and Skaalvik, 2016), and there is also previous evidence showing that teachers’ experience of high levels of effort is related to high levels of emotional exhaustion (Wang et al., 2015). Based those findings, it was reasonable to expect that profile groups identified on a basis of work engagement, along with work-related effort and reward would have differed with respect to teachers’ experiences of emotional exhaustion as well. However, regarding to profile groups 2 and 3, the lack of difference in teachers’ emotional exhaustion can perhaps be explained with the lack of difference in teacher-reported occupational stress. This is because the previous literature has shown that an experience of emotional exhaustion builds on the experience of prolonged stress (e.g., Maslach et al., 2001; Schaufeli and Salanova, 2013). Therefore, as there was no statistically significant difference between the profile groups 2 and 3 with respect to occupational stress, it is understandable that there is no significant difference in teachers’ experience of emotional exhaustion either. Perhaps this explanation is somewhat applicable to the non-significant (*p* = 0.73) difference in teachers’ emotional exhaustion between the profile groups 1 and 3 as well.

Third, as expected (Hypothesis 2b), there were some differences among the profile groups with respect to self-reported changes in occupational well-being due to the COVID-19 pandemic. Based on the findings concerning both of the measured indicators (i.e., the extent to which the increase in teachers’ occupational stress was related to the COVID-19 pandemic and a change in emotional exhaustion), the occupational well-being of teachers in profile group 3 (i.e., *Highly Engaged with Higher Reward than Effort*) seemed to be less influenced by the COVID-19 pandemic than it was in the other two profile groups. However, profile groups 1 and 2 did not differ with respect to change in occupational stress nor with respect to change in emotional exhaustion. Nevertheless, these findings are also in line with previous studies suggesting that an experience of work engagement and different job-related resources may protect against the negative impacts of job-related demands and the other elements that negatively affect well-being (e.g., Bakker et al., 2007). While the present study cannot be used to determine the critical point where this possible protection process may begin, it is important to notice how the strains that teachers are facing with the COVID-19 pandemic and will surely have to face in future pandemics, can be supported by strengthening teachers’ work engagement as well as by increasing the balance between experiences of work-related effort and reward.

Fourth, in contrast to what was expected (Hypothesis 2c), the profile groups did not clearly differ with respect to changes in work-related effort and reward due to the COVID-19 pandemic. Understandably, profile group 1 (i.e., *Poorly Engaged with Highest Effort and Lowest Reward*) and profile group 2 (i.e., *Averagely Engaged with Higher Effort than Reward*) did not differ with respect to changes of work-related effort and reward due to the COVID-19 pandemic, as those profile groups also did not differ with respect to work-related effort and reward when used as criterion variables for the profile analysis. The only statistically significant difference showed that teachers recognized as being *Highly Engaged with Higher Reward than Effort* (i.e., profile group 3) reported a higher increase in their work-related effort than teachers recognized as being *Averagely Engaged with Higher Effort than Reward* (i.e., profile group 2). In other words, teachers with lower levels of work-related effort than reward were the ones who felt that their work-related effort had actually increased more due to the COVID-19 pandemic than teachers with higher levels of work-related effort than reward. Unfortunately, the cross-sectional data used for the present study do not allow one to examine the change in more detail. However, the findings related to Hypothesis 2c can be seen to indicate two things. First, it should be noted that in the present sample, teachers’ experiences of change in their work-related reward due to the COVID-19 pandemic did not differ between profile groups. That seems quite a reassuring finding, as it means that despite evident changes and job-related demands as a result of the COVID-19 pandemic (e.g., Kim and Asbury, 2020; MacIntyre et al., 2020), teachers in different profile groups did not differ in terms of how they felt about changes in receiving adequate support and appreciation from their peers and leaders. Second, similar to what was discussed above, these findings highlight that an experience of work engagement and different job-related resources may provide protection against the negative impacts of job-related demands and the elements that are harming the well-being (e.g., Bakker et al., 2007; Bermejo-Toro et al., 2016). Teachers in profile group 3 (i.e., *Highly Engaged with Higher Reward than Effort*) experienced an increase in their effort due to the COVID-19 pandemic, yet they still had the lowest levels of effort when it was used as a criterion variable for the profile groups. Thus, the occupational well-being of these teachers was not perhaps severely affected by this increase of effort; instead, they were able to cope well with the situation.

The third research question focused on the extent to which the identified profile groups differed with respect to the self-reported individual resource of work meaningfulness and the leader-level resource of the leader–follower relationship. The findings suggest, as proposed in Hypothesis 3a, that each profile group differed based on the self-reported individual resource of work meaningfulness. Teachers recognized as being *Highly Engaged with Higher Reward than Effort* (i.e., profile group 3) reported the highest work meaningfulness, while teachers recognized as being *Poorly Engaged with Highest Effort and Lowest Reward* (i.e., profile group 1) reported the lowest. These findings are important for at least the following reasons. First, as prior research has indicated that teachers’ experience of work meaningfulness is positively related with their work engagement (e.g., Ugwu and Onyishi, 2018) and that individual resources such as a sense of work’s meaningfulness helps a worker to cope with the demands of the job (e.g., Minkkinen et al., 2020), the findings showing that each profile group differed with respect to work meaningfulness can be seen to validate the current three-profile solution. Second, the findings highlight the importance of individual resources of work meaningfulness. Particularly when considered along with the findings gained with respect to Hypothesis 2c, these findings can also be seen to concur with a prior finding suggesting that a sense of meaningful work protects teachers’ well-being under stressors (Minkkinen et al., 2020). Perhaps these findings can be interpreted to imply that a sense of work meaningfulness should be seen as a critical factor during this time of recovery from the COVID-19 pandemic and beyond. Moreover, it should be remembered that teachers need to experience their job as meaningful, and perhaps particularly so when they are working under exceptional circumstances for some reason. Therefore, it would be important to examine more, for example, the ways in which the COVID-19 pandemic and the different restrictions that resulted from it have influenced teachers’ social relations with other teachers, parents, and students, and whether any changes that occurred have impacted teachers’ sense of work meaningfulness.

Finally, somewhat in line with what was expected (Hypothesis 3b), there were some differences with respect to the leader-level resource of the leader–follower relationship among the profile groups. Teachers recognized as being *Highly Engaged with Higher Reward than Effort* (i.e., Profile group 3) reported a higher quality of leader–follower relationship than teachers recognized as being *Averagely Engaged with Higher Effort than Reward* (i.e., profile group 2). While no other differences were found, the findings concur with the previous ones (e.g., Collie, 2021) by indicating that for some teachers, the quality of the leader–follower relationship can be seen as related to their occupational well-being. Perhaps it could even be possible to speculate whether by enhancing the leader-level resource of the quality of leader–follower relationship, teachers recognized as being *Averagely Engaged with Higher Effort than Reward* (i.e., profile group 2) could establish one beneficial buffer against the work-related demands, which could enhance their occupational well-being further (see Christian et al., 2011; Nielsen et al., 2017; Lesener et al., 2020). Yet, the present findings with respect to the absence of differences in the leader–follower relationship among the profile groups can raise the question of whether teachers recognized as being *Poorly Engaged with Highest Effort and Lowest Reward* (i.e., profile group 1) can efficiently benefit the quality of the leader–follower relationship as a job-related resource. The fact that there was no difference in the leader–follower relationship between profile groups 1 and 3 suggests that teachers recognized as being *Highly Engaged with Higher Reward than Effort* (i.e., profile group 3) were perhaps more capable of utilizing the support gained from leaders than teachers recognized as being *Poorly Engaged with Highest Effort and Lowest Reward* (i.e., profile group 1). Thus, more research is needed to find ways to enhance the occupational well-being of all teachers, for example, by focusing on ways the leader can provide such feedback that enhances teachers’ work engagement during exceptional times and in other ways as to how teachers can be supported *via* social capital integrated in schools.

## Limitations and Suggestions for Future Studies

This study has some limitations. First, the measures used to capture the possible change in teachers’ emotional exhaustion as well as in work-related effort and reward due the COVID-19 pandemic were used for the first time in the current study. While the measures were adapted by adding simple questions along with the original measures (i.e., the original subscale of emotional exhaustion of the Finnish version of the BBI, Salmela-Aro et al., 2011, and the original subscales of effort and reward of the ERI scale, Siegrist et al., 2004), and the internal consistencies for the adapted measures were acceptable, it would be important to use the same measures in future studies to gain more experience on applicability of these measures. Second, the data used for the present study were cross-sectional, and, thus, based on the data used, no causal inferences can be made. In addition, results with respect to changes in teachers’ experiences due to the COVID-19 pandemic were obtained by asking teachers to consider whether their experiences had remained relatively the same or whether there was an increase or a decrease when compared with the time before the COVID-19 pandemic. It is possible that such questions are not easy to evaluate. These kinds of limitations could be faced if there would be a possibility to collect longitudinal data that could capture possible changes in teachers’ experiences of their occupational well-being repeatedly and simultaneously while the COVID-19 pandemic evolves. Such data could also allow examination of causal directions. Finally, it is evident that the number of teachers in profile group 1 was rather small (*n* = 15). While there was no reason to doubt the reliability of the present results due to analytical approaches that were chosen, one should consider the size of the group when generalizing the findings as the group covers only limited number of teachers. While the percentage of teachers with the poorest occupational well-being is mercifully quite low in general, it also means that the total number of participants should be relatively large in order to reach bigger number of participants recognized as having the poorest well-being.

The findings gained in the present study provide some suggestions for future studies. First, as the present findings align with the prior ones (e.g., Herman et al., 2021; Pöysä et al., 2021) by showing that teachers’ occupational well-being is individually constructed, they suggest that in order to enhance the theory development and provision of practical suggestions, future studies should be conducted using analytical approaches that move beyond traditional variable-oriented approaches. Such studies are needed, for example, to examine further the differences in the ways in which teachers’ work engagement is built on the basis of job-related and personal resources. Using a person-oriented approach with a larger dataset would provide insightful and valuable knowledge on what kind of support would be beneficial for teachers with different well-being profiles. This kind of understanding would be central during the time of the COVID-19 pandemic as well as in order to prepare for possible future crises. In addition, while the present findings complimented the literature and provided a relatively practical perception that there are differences in the ways in which certain job-related resources, for instance, a leader-level resource of leader–follower relationship, may be related to teachers’ occupational well-being, more research is needed to gain a deeper understanding of this phenomenon. It would be important to find the ways in which each teacher could benefit from resources integrated into the social capital of the teachers’ work environment.

## Data Availability Statement

The datasets presented in this article are not readily available because of the ongoing research. Requests to access the datasets should be directed to sanni.poysa@jyu.fi.

## Ethics Statement

The studies involving human participants were reviewed and approved by the ethical committee of the University of Jyväskylä. Written informed consent for participation was not required for this study in accordance with the national legislation and the institutional requirements.

## Author Contributions

SP was responsible for the research questions and statistical analyses of the present manuscript, and she functioned as a corresponding author. EP was the Responsible Researcher and M-KL was the Principal Investigator of the larger Teacher and Student Stress and Interaction in Classroom study (TESSI; Lerkkanen and Pakarinen, 2021), under which the current study was conducted. They were responsible for the design, data collection, and publication plan of the TESSI study, and together they supported SP in analyzing the data and coauthoring the current manuscript. All authors contributed to the article and approved the submitted version.

## Funding

This study is a part of the project EduRESCUE–the resilient schools and the education system. This study was funded by the Strategic Research Council (SRC) established within the Academy of Finland (345196), by grants from the Academy of Finland (nos. 335635 and 317610), and by the grant from The Trade Union of Education in Finland.

## Conflict of Interest

The authors declare that the research was conducted in the absence of any commercial or financial relationships that could be construed as a potential conflict of interest.

## Publisher’s Note

All claims expressed in this article are solely those of the authors and do not necessarily represent those of their affiliated organizations, or those of the publisher, the editors and the reviewers. Any product that may be evaluated in this article, or claim that may be made by its manufacturer, is not guaranteed or endorsed by the publisher.
